# A study of COVID anxiety, spiritual well-being and resilience levels in patients with cancer undergoing chemotherapy during the COVID-19 pandemic: a cross-sectional study in the south of Iran

**DOI:** 10.1186/s40359-023-01126-1

**Published:** 2023-03-20

**Authors:** Zahra Khiyali, Zeinab Naderi, Mohammadkazem Vakil, Hajar Ghasemi, Azizallah Dehghan, Mostafa Bijani

**Affiliations:** 1 Department of Gerontology, University of Social Welfare and Rehabilitation Science, Tehran, Iran; 2 Department of Nursing, Sirjan School of Medical Sciences, Sirjan, Iran; 3 Noncommunicable Diseases Research Center (NCDRC), Fasa University of Medical Sciences, Fasa, Iran; 4 Department of Medical Surgical Nursing, School of Nursing, Fasa University of Medical Sciences, Fasa, 81936-13119 Iran

**Keywords:** COVID-19, Spirituality, Health, Resilience, Anxiety, Patients

## Abstract

**Background:**

Patients with cancer are at higher risk of contracting COVID-19 with poor prognosis. Therefore, the present study was conducted to investigate anxiety, spiritual well-being, and resilience levels in patients with cancer undergoing chemotherapy during the COVID-19 pandemic in the south of Fars Province, Iran.

**Methods:**

This is a descriptive study with a cross-sectional design conducted on the patients undergoing chemotherapy at Dr. Ali Shariati Hospital in Fasa from November 2021 to February 2022. Cancer patients undergoing chemotherapy were included in the study by census method. Out of 210 patients, 155 participated in the study. Data were collected electronically using the standard instruments of Ellison’s Spiritual Well-being Scale, COVID-19 anxiety questionnaire, and Connor-Davidson resilience scale. The collected data were analyzed in SPSS 22 using descriptive statistics, Pearson correlation tests, T-test, ANOVA and multivariate linear regression at a level of significance of *P* < 0.05.

**Results:**

The participants’ resilience mean score was 46.35 ± 26.51 and their spiritual well-being mean score was 69.58 ± 9.32. In addition, their COVID anxiety mean, score was found to be 16.85 ± 10.51. The results showed a significant direct correlation between the patients’ spiritual well-being and resilience (r = 0.47, *P* < 0.001) and a significant inverse correlation between the patients’ spiritual well-being and COVID-19-related anxiety (r = − 0.275, *P* < 0.001). In addition, there was a significant inverse correlation between the variables of resilience and COVID-19-related anxiety (r = − 0.637, *P* < 0.001). Based on multivariate linear regression, the most common predictors in resilience were age and history of infection with COVID-19, and in spiritual health and anxiety, was a history of infection with COVID-19.

**Conclusion:**

Enhancement of spiritual well-being and resilience in patients should be an integral part of care as these qualities are valuable resources in fighting cancer and lowering patients’ anxiety, especially during the COVID-19 pandemic.

## Introduction

As a new strain of the coronavirus family, COVID-19 was first seen in late December 2019, in some individuals in Wuhan, China, who were suffering from severe pulmonary infection. The disease quickly spread across the world [1]. The infectiousness of COVID-19 was so high that on March 11, 2020, World Health Organization (WHO) declared it a pandemic [2]. According to global statistics, the fatality rate of the disease is 3.4%, but that figure varies according to patients’ ages and health conditions, including the presence of underlying diseases [3]. COVID-19 has caused great tension in providing healthcare services and directly or indirectly affected the treatment procedures for many common diseases. During the current pandemic, patients with cancer are among the high-risk groups because of the deficiencies in their immune system caused by their illness and exposure to different cancer treatments [4]. Because of the issues associated with their underlying illness and systematic suppression of their immune system following deterioration in their health condition and administration of chemotherapy or surgeries, patients with cancer are at high risk of a variety of traumas and disorders caused by other illnesses. The risk of acute conditions in patients with cancer is considerably higher than in the general population, thus this group of patients is at higher risk of contracting COVID-19 with poor prognosis [5, 6]. Research shows that patients with cancer are at greater risk in terms of contracting more severe infections and the consequent complications, especially if they undergo surgery or chemotherapy one month before being infected by the new coronavirus [4, 7]. These conditions have been found to cause significant increase in the anxiety and mental and physical stress levels in patients with cancer [8]. COVID-19 anxiety can influence the decisions of patients with cancer regarding seeking treatment or continuing their treatment. These patients have faced many challenges and stressful situations since the onset of the pandemic, which, in some cases, have disinclined them to visit the hospital and pursue their treatment [9]. The studies of Vicinanza et al. [10] and Grajek et al. [11], highlights the presence of clinically significant anxiety in the cancer patients undergoing radiotherapy during the pandemic of COVID-19.

Studies by psychologists have shown that there are moderating factors between stressful events and psychological disorders which influence the impact of these events on different individuals, and one of these factors is spiritual well-being [12].

Spiritual well-being is one of the dimensions of well-being that creates consistency between the other dimensions. An increasing number of health experts believe that spirituality is an influential factor in curing many psychological, physical, and mental hygiene disorders [13]. Spiritual well-being is comprised of religious well-being and existential well-being. Religious well-being refers to having a connection with God, an infinite source of power, while existential well-being concerns our relationships with others, our environment, and our inner self [14]. Studies show that religion and spirituality can play a major part in patients’ coping with cancer, protecting their mental health, and improving the quality of their lives. In addition, religious/spiritual adaptation is regarded as a very beneficial strategy for patients who are on chemotherapy, especially in the long run [15].

Another important factor in improving mental health and reducing anxiety and stress in patients is resilience. Resilience is the ability to improve one’s social performance and overcome adversities in the face of great stress and dangerous conditions [16]. Resilience is a quality that helps individuals deal with and adapt to stressful life events and protects them against psychological disorders and obstacles. Resilient individuals possess good coping capacity that enables them to deal with environmental stressors [17]. These individuals are equipped with good self-regulation or self-control skills, a positive self-image, high autonomy and self-esteem, and good communication and problem-solving skills that make a significant contribution to their mental health [18]. According to a study by Yamile et al. investigating resilience in patients with cancer, cancer affects all the aspects of the lives of the patients and a large part of the issues that the patients are faced with are emotional and mental problems. Therefore, studies of resilience, which is an integral component of psychological care for patients, can underscore the positive results of enhancing patients’ resilience before, during, and after cancer treatment [19].

Most of the previous work on linkages between distress, resilience and spiritual well-being in patients undergoing chemotherapy during the COVID-19 Pandemic was conducted in developed countries and different cultural characteristics; these findings may not be generalizable to developing countries with different population characteristics [2–21]. Moreover, the simultaneous examination of the relationship between the three variables of spiritual well-being, anxiety and resilience in patients under Chemotherapy has not yet been studied in Iran.

On the other hand, studies have shown that culture and cultural values can indirectly affect the level of anxiety and stress that people perceive in stressful situations [22] and they can be effective on the spiritual health of people [23]. Cancer, like other diseases, is not exempt from the influence of cultural factors. Cultural aspects, values and behaviors along with life experiences, socio-economic status and personality differences determine the meaning of cancer for patients and their families, and it affects how they deal with the disease, the continuation of the treatment, the management of complications and the prognosis of the disease [24, 25].

Therefore, the present study was conducted to investigate anxiety, spiritual well-being, and resilience levels in patients with cancer undergoing chemotherapy during the COVID-19 pandemic in the south of Fars Province, Iran.

## Methods

**Fig. 1:**
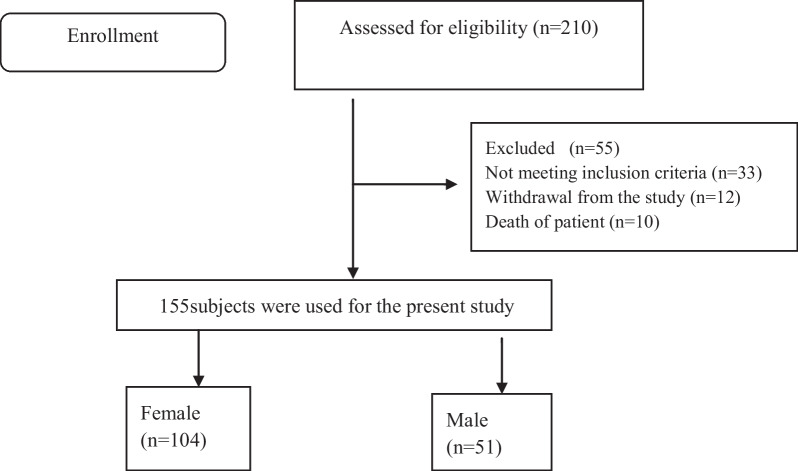
Participant recruitment flow diagram

This is a descriptive study with a cross-sectional design conducted on the patients undergoing chemotherapy at Dr. Ali Shariati Hospital in Fasa from November 2021 to February 2022. Dr. Ali Shariati Hospital is located in Fasa, in the south of Fars province. The oncology department of this hospital is equipped with 22 beds and accommodates 210 patients. Cancer patients undergoing chemotherapy were included in the study by census method. Out of 210 patients, 155 participated in the study and 55 did not participate in the study for various reasons (Fig. 1). The inclusion criteria were being over 18, being completely conscious, having at least reading and writing literacy, and not having a known psychological disorder. The patients who did not complete the questionnaire or completed the questionnaire partially were excluded from the study.

### Data collection

During the COVID-19 pandemic, the oncology department was operational and provided care to patients. A nurse from the oncology department of the hospital where the study was conducted (author 4) explained the objectives and method of the study to the patients. The patients who consented to participate were asked to complete the electronic version of the questionnaires that were submitted to them via their cell phones or tablets allocated for this research. The questionnaires were completed on a self-report basis by the patients, who were asked to give honest answers to the questions. All queries were dealt with by the researcher.

### Data collection instruments

#### Ellison’s spiritual well-being scale

This scale consists of 20 questions: 10 questions address religious well-being and 10 questions address existential well-being. The score range of each of the religious and existential sections is from 10 to 60. A respondent’s spiritual well-being score is the sum of these subcategories and ranges from 20 to 120. A score of 20–40 indicates low spiritual well-being, 41–99 indicates moderate spiritual well-being, and 100–120 indicates high spiritual well-being [26]. The validity and reliability of this scale has been verified in a study by Seyedfatemi et al. who found the Cronbach’s alpha coefficient of the scale to be 0.82 [27].

#### COVID-19 anxiety questionnaire

Developed by Alipour et al. [28], this questionnaire consists of 18 questions that measure COVID-19 anxiety on a 4-point Likert scale, ranging from never, occasionally and usually to always. Higher scores indicate greater anxiety in the respondents. A score of 0–16 indicates lack of anxiety or mild anxiety, 17–29 indicates moderate anxiety, and 30–54 indicates severe anxiety. The content validity of the questionnaire was verified by five experienced psychologists who evaluated the questions in terms of clarity and whether they addressed all the aspects of the subject and the appearance of the instrument. The reliability of the questionnaire was confirmed by a Cronbach’s alpha of 0.91.

#### Connor–Davidson resilience scale

Developed by Connor and Davidson in 2003, this scale consists of 25 items that fall into five subscales: personal competence and trusting one’s instincts, handling negative emotions, having a positive attitude to change and secure relationships, control, and spirituality. The items are scored on a 5-point Likert scale: not true at all = 0, rarely true = 1, sometimes true = 2, often true = 3, and true all of the time = 4. The score range is between 0 and 100. The cut-off point of the scale is 50; thus, a score of above 50 indicates high resilience, and the higher a respondent’s score is, the greater his/her resilience is and vice versa. In this scale, a score of 0–33 shows unfavorable resilience, 34–67 indicates moderate resilience, and 68–100, indicates favorable resilience. Connor and Davidson reported the reliability of the scale to be 0.71 [29]. In a study by Haji et al. [30], the reliability of the Persian version of the scale was found to equal a Cronbach’s alpha of 0.77, and the validity of the scale was verified by a panel of experts.

### Ethical considerations

All the participants gave written informed consent to participate in the study. The present study was conducted in terms of the principles of the revised Declaration of Helsinki, which is a statement of ethical principles that directs physicians and other participants in medical research involving human subjects. The participants were assured about their anonymity and confidentiality of their information. Moreover, the study was approved by the Institutional Research Ethics Committee of Fasa University of Medical Sciences, Fasa, Iran (Ethical Code: IR.FUMS.REC.1400.087).

### Data analysis

The collected data were analyzed in SPSS v. 22 using descriptive statistics, Pearson correlation tests, T-test, ANOVA and multivariate linear regression. Level of significance was set at *P* < 0.05.

## Results

In total, 155 patients participated in the study, 67% of whom were male. The mean of the participants’ ages was 63.50 ± 15.89 years and the mean of their illness duration was 2.12 ± 2.15 years. The most common types of cancer among the patients were, in order of prevalence, stomach cancer (14.20%) and breast cancer (13.50%). The other demographic characteristics of the patients are shown in Table 1.

**Table 1:** Frequency distribution of the demographic characteristics of the participating patients (N = 155)

Variables	Number	Percentage
*Gender*		
Female	104	67
Male	51	33
*Education*		
Elementary	41	26.50
high-school	49	31.60
diploma	55	35.5
More than diploma	10	6.4
*Marital status*		
Single	14	9
Married	141	91
*Job*		
Home maker	50	32.3
Employee	4	2.6
Self-employed	89	57.4
Retired	9	5.8
Unemployed	3	1.9
*History of infection with COVID-19*		
Positive	72	46.50
Negative	83	53.50

The participants’ resilience mean score was 46.35 ± 26.51. Their spiritual well-being mean score was 69.58 ± 9.32, with their mean scores for the subscales of religious well-being and existential well-being equaling 35.04 ± 5.49 and 34.54 ± 4.91, respectively. In addition, their COVID anxiety mean score was found to be 16.85 ± 10.51, with their mean scores for the subscales of mental and physical anxiety being 11.69 ± 6.54 and 5.61 ± 4.84, respectively. Table 2 shows the frequency distribution of the variables of resilience, spiritual well-being, and COVID anxiety per division.

**Table 2:** Frequency distribution of the resilience, spiritual well-being, and COVID anxiety of the participating patients (N = 155)

Variables	Number	Percentage
*Resilience*		
Un favorable	47	30.30
Moderate	72	46.50
Favorable	36	23.20
*Spiritual well-being*		
Low	1	0.6
Moderate	152	98.10
High	2	1.30
*COVID anxiety*		
Mild	65	41.90
Moderate	74	47.70
Severe	16	10.30

The results of the partial correlation test in Table 3 showed that there was a positive direct correlation between spiritual well-being and resilience (r = 0.47, *P* < 0.001) and an inverse correlation between spiritual well-being and COVID anxiety (r = − 0.275, *P* < 0.001). In addition, there was an inverse correlation between the variables of resilience and COVID anxiety (r = − 0.637, *P* < 0.001).

**Table 3:** Relationship between resilience, spiritual well-being, and COVID anxiety of the participating patients (N = 155)

Variable		*P* value	r
Spiritual well-being	Resilience	< 0.001	0.47
Spiritual well-being	COVID anxiety	< 0.001	0.275
Resilience	COVID anxiety	< 0.001	0.637

Based on the results shown in Table 4, the females, retired patients, and patients with a negative history of infection with COVID-19 had significantly higher spiritual well-being than the other patients. On the other hand, anxiety levels were significantly higher in the male patients and the patients with a negative history of infection with COVID-19. The females, self-employed patients, and patients with a positive history of infection with COVID-19 had significantly higher resilience. In addition, only the variable of resilience was found to have an inverse relationship with age (r = − 0.319, *P* < 0.001), and the variables of spiritual well-being and anxiety did not have a relationship with age. Based on the results of the multivariate linear regression demonstrated in Table 5, the most common predictors in resilience were age and history of infection with COVID-19; accordingly, increased age and having a history infection with COVID-19 were related with a decrease in resilience score. In addition, a history of infection with COVID-19 was the most common predictors in spiritual health and anxiety; thus, having a history of corona disease was related with a decrease in anxiety and spiritual scores.

**Table 4:** The correlation between the variables of resilience, spiritual well-being, and COVID anxiety and the demographic characteristics of the participating patients

Qualitative variables	Resilience			Spiritual well-being			COVID anxiety		
	M	SD	*P*	M	SD	*P*	M	SD	*P*
*Gender*									
Female	59.16	24.90	< 0.001*	71.55	8.22	< 0.001*	14.38	11.75	< 0.001*
Male	39.31	24.78		66.01	10.21		18.21	9.54	
*Education*									
Semi-literate	44.75	24.98	0.714**	69.85	10.08	0.643**	18.90	12.94	0.264**
Some high-school	53.44	24.45		69.06	8.47		16.04	9.14	
High-school diploma	47.71	29.23		69.21	8.02		16.92	9.77	
More than high-school	55.4	23.80		73.10	15.96		12	8.61	
*Marital status*									
Single	56.07	26.84	0.235*	66.69	19.35	0.608	18.38	15.79	0.151*
Married	40.11	26.14		69.91	7.89		16.82	9.89	
*Job*									
Home maker	58.06	25.28	< 0.001**	65.9	10.63	< 0.001**	14.68	11.65	0.255**
Employee	64.50	27.59		65	3.55		12.5	10.75	
Self-employed	68.33	22.94		65.04	6.24		11.33	9.71	
Retired	36.36	23.22		72.5	8.03		18.41	9.09	
Unemployed	64.77	23.92		64.77	6.15		16.77	15.54	
*History of infection with COVID-19*									
Positive	58.45	24.38	< 0.001*	67.66	11.24	0.006*	13.86	10.74	< 0.001*
Negative	32.4	21.67		71.8	5.81		20.29	9.14	

*Independent T-test

**One-way variance analysis test (ANOVA)

**Table 5:** Multivariable Linear Regression Models for resilience, spiritual well-being, and COVID anxiety of the participating patients (N = 155)

	Resilience			COVID anxiety			Spiritual well-being		
	β	SE	*P*	β	SE	*P*	β	SE	*P*
Age	− .354	.115	.002	.003	.055	.959	.026	.058	.652
Gender	10.170	8.219	.218	− 1.235	3.958	.755	− 2.598	3.536	.464
Job	.073	2.678	.978	.609	1.291	.638	.779	1.152	.500
History of infection with COVID-19	− 23.045	3.505	.000	− 6.202	1.669	.000	− 3.285	1.507	.031
Married	5.775	5.965	.335	–	–	–	1.328	2.567	.606
Education							.659	.837	.432

## Discussion

The present study was conducted to investigate COVID anxiety, spiritual well-being, and resilience levels in patients undergoing chemotherapy during the COVID-19 pandemic. The findings showed that the majority of the patients had average spiritual well-being. Probably, this can be related to the fact that the people of Iran are religious according to the cultural conditions and beliefs of the people, and they turn to religion more to adapt to the crisis-causing conditions. The level of spiritual well-being was significantly higher in the female patients, retired patients, and patients with a negative history of infection with COVID-19. According to Coppola et al. [31], the higher scores of women's spiritual well-being could be linked to the fact that women have different experiences and coping strategies to men and to the fact that religious norms and beliefs are more compatible with roles, characteristics and behaviors socially attributed to women. On a similar note, in studies by Sun et al. [32] and Martins et al. [33], gender had a significant impact on the spiritual well-being of patients with cancer who were undergoing chemotherapy: the females obtained higher spiritual well-being scores than the males. Moreover, the majority of the patients had average levels of spiritual well-being. In a study by Li et al. [34], 69% of the Taiwanese patients with colorectal cancer and history of colostomy had average levels of spiritual well-being. In their study, Amirmohamadi et al. [35] reported that patients with cancer possessed average spiritual well-being and the subscale of religious beliefs received the highest mean score. However, Kavak et al. [36] found that patients with advanced gastric cancer had high levels of spiritual well-being and their highest mean score belonged to the subscale of religious beliefs. This discrepancy can be due to differences in the study settings and the type and stage of cancer addressed by the researchers.

In the present study, there was not a statistically significant correlation between the variables of age, marital status, and education on the one hand and spiritual well-being on the other, which is consistent with the findings of other studies [21, 33]. According to some studies, there is no correlation between spiritual well-being and the general variables of age, gender, race, stage of cancer, metastasis, medical insurance, marital status, religious tendencies, etc. [37]. On the contrary, some other studies report that the demographic characteristics and clinical data (e.g. age, gender, race, tumor type, cancer location, occupation, religious beliefs, etc.) of patients with advanced cancer influence the patients’ spiritual well-being and that older patients possess better spiritual well-being [32].

The findings of the present study showed that the majority of the patients had average levels of COVID-19-related anxiety. Nevertheless, anxiety levels were significantly higher in the male patients and the patients with a negative history of infection with COVID-19.

In a study by Dehghan et al. [38], 61.4% of the patients with intermediate to advanced stages of cancer reported that they were mentally and physically anxious about COVID-19, and 26.1% believed that the spread of COVID-19 and the conditions and obstacles associated with it had affected the process of their treatment and caused challenges in their lives. Similarly, according to a study by Kim et al. [39], in the COVID-19 pandemic, patients with cancer experienced depression and anxiety about the viral pandemic and disruption to their access to healthcare services, which may have adversely affected their mental health and led to behavioral disorders. The studies of Vicinanza et al. [10] and Grajek et al. [11], highlights the presence of anxiety in the cancer patients undergoing radiotherapy during the pandemic of COVID-19. Also, the findings of previous studies showed that the side effects of chemotherapy can cause psychosocial damage such as pain, depression, insomnia and anxiety in patients [40, 41] and during the epidemic of Covid-19, consequently increased psychological pressure due to COVID-19, was increased the level of these psychological damages [42]. Concerns about contracting or a family member contracting COVID-19 are also strongly related to anxiety and generate depressive behaviors [43], and the increased fear related to the contraction of the virus and the fact that family members have contracted the infection directly affects all these emotional aspects [44]. Due to the critical condition of COVID-19, cancer patients’ communication with the community and their health care professionals, who convey accurate information about the disease has decreased. The current state of the COVID-19 epidemic has also made it difficult for doctors to make decisions. This condition causes anxiety in cancer patients. Also, for patients with a negative history of infected with COVID-19, the fear of being infected with the corona virus brings a lot of anxiety, so that anxiety and fear of being infected with the corona virus is one of the most common reasons that leads to a delay in treatment [45]. In contrast to the findings of the present study, Sigorski et al. [45], Mungase et al. [46], Rassoulian et al. [47] showed that women with cancer experienced anxiety significantly more than men. This discrepancy can be due to differences in the instruments used by the researchers, environments and the cultural ground dominating this city including people's patience and tolerance, especially the women of this city in dealing with the problems, adaptation processes.

In the present study, the majority of the patients were found to possess average levels of resilience. It was also found that the female patients, self-employed patients, and patients with a positive history of infection with COVID-19 had significantly higher resilience. This level of resilience in women can be caused by the high level of spiritual health of women and their ability to tolerate and adapt to problems and difficult conditions, which is considered one of the cultural characteristics of women in this city [48]. In line with the present study, the studies of Kavak et al. [36], Gao et al. [49], reported the moderate level of resilience in patients with oral cancer and gastrointestinal cancer, respectively.

Based on previous studies, there is a positive correlation between gender and resilience [36, 50], with the resilience score of females lower than that of males [36]. Moreover, there was a moderate inverse correlation between resilience and age. For the relationship between age and resilience, there were studies that reported both positive [51] and negative correlations [52, 53]. Montazeri's et al. [54], reported that resilience has a significant relationship with the history of Covid-19 in the group of men. In addition, men's unemployment and women's staying at home reduce the level of resilience. In the present study, the results showed a moderate direct correlation between spiritual well-being and resilience in the cancer patients undergoing chemotherapy.

Recent research points to the role of spirituality in promoting resilience in different ways inluding favoring interpersonal relationships, as a source of strength and inner peace, or reducing feelings of anger and social isolation. It shows that spiritual well-being is associated with resilience [50–55]. According to a study by Kavak et al. [36], in patients with advanced gastrointestinal cancer, higher levels of spirituality correlated with greater psychological resilience; the researchers concluded that the patients might rely on spiritual coping strategies to come to terms with the undesirable consequences of their illness. The findings of a study by Fradelos et al. [15] showed a strong correlation between religiosity and resilience in patients with breast cancer, so that religion can act as a mechanism that gives meaning to life, especially at the time of stressful events. Thus, religiousness contributes to resilience and helps individuals maintain a positive outlook during adversities.

The findings of the present study showed an inverse correlation between spiritual well-being and COVID-19-related anxiety in the cancer patients undergoing chemotherapy. Mihic-Gongora et al. found a negative correlation between psychological distress, spirituality and resilience in people with advanced cancer during the COVID-19 pandemic. The results established that spirituality played a 12.1% mediating role [20]. In the study of Turke et al. [56], the significant inverse correlation between anxiety levels and spirituality suggested that spirituality in patients with cancer decreased anxiety levels during social isolation and helped patients to face cancer treatment during the COVID-19 pandemic. Moeini et al. found that a spiritual care plan could successfully reduce anxiety levels in patients with Leukemia. Thus, in the case of hard-to-treat diseases, like cancer, nurses are advised to use holistic care plans that stress spiritual care. While patients’ spiritual needs are often ignored, satisfaction of hospitalized patients’ spiritual needs is crucial for speeding up their recovery, acquiring spiritual health, and controlling their anxiety [57].

Even though most of the literature reports a positive correlation between spirituality and mental health, some studies have found the correlation between the two to be negative or insignificant [44]. In a study by Dehghan et al. [38], the results showed no correlation between COVID-19-related anxiety and spiritual well-being. In this study, cancer patients had good spiritual health (27.7%: high and 71.7%: moderate), and the high spiritual health of the study population may not determine the relationship between spiritual health and coronavirus anxiety.

Another noteworthy finding of the present study was the presence of an inverse correlation between the variables of resilience and COVID-19-related anxiety in the patients undergoing chemotherapy. Similarly, an increasing body of literature shows that, both in cancer patients and cancer survivors, resilience correlates with better adaptation to cancer, higher quality of life, better mental health, and improved treatment results [58]. In a study by Javellana et al. [59], higher levels of resilience in women with ovarian cancer predicted higher health-related quality of life scores and less anxiety about the COVID-19 pandemic. On a similar note, Koral and Cirak (2021) reported a significant inverse correlation between fears of cancer recurrence on the one hand and spiritual well-being and psychological resilience on the other in breast cancer survivors. Despite their failure to follow up on their treatment due to the spread of COVID-19, breast cancer survivors with higher spiritual well-being and psychological resilience scores were found to experience less fear of cancer recurrence. In addition, there was a significant positive correlation between psychological resilience and spiritual well-being in the patients [60].

Similarly, in studies by Mungase et al. [46] and Gao et al. [49], the results showed a significant inverse correlation between resilience and anxiety in cancer patients who were undergoing radiotherapy. The results of a study by Tamura et al. [50] showed that resilience levels predicted the extent of anxiety, depression, and quality of life of patients with colorectal cancer who were on chemotherapy. Thus, enhancing resilience in these patients is crucial for improving their mental health and quality of life.

Greater resilience does not provide immunity from emotional anxiety, but it enables individuals to maintain their emotional stability beyond negative personal experiences and difficult environmental conditions [61]. Accordingly, development of resilience mechanisms in the course of cancer treatment can help improve the patients’ mental health and quality of life [50].

The findings of the present study showed that the most common predictors in resilience were age and history of infection with COVID-19, so that increased age and having a history infection with COVID-19 were related with a decrease in resilience score. In addition, history of infection with COVID-19 was the most common predictors in spiritual health and anxiety. Thus, having a history of corona disease was related with a decrease in anxiety and spiritual scores. In line with the present study, the results obtained from the study by Ferreira et al. [62] showed that elderly people and those who had a person infected with Covid-19 in their family and surroundings were less resilient. Study of Kordan on cancer patients showed that resilience decreases with age [63]. Greater weakness and vulnerability (comorbidities, lower performance) and dependency, and poorer tolerance to cancer treatment in the elderly account for this result [20]. Oppositely, an exploratory cross-sectional study on association of resilience and age in individuals with colorectal cancer revealed older patients reported higher resilience [64]. The study is inconsistent with our study. Considering the large proportion of men in our study, our results can be attributed to the fact that with increased age, responsibility increases, and psychological, social, economic, and personal burden of disease increase, consequently psychological well‑being, social capital, and resilience decreases.

In contrast to the results of the present study, Moarrefzadeh and et al. [65] reported that the history of infection with coronavirus could increase the severity of anxiety and have a more negative effect on mental health. In crisis conditions, the social and individual structures of life face chaos. People feel that the amount of control they have over the course of life has been reduced, and this situation gives rise to a feeling of insecurity and this lack of security will cause anxiety. This conflict can be answered by considering the strength of the family center and the comprehensive support of families to their patients in the cultural context of Fasa city and the fact that the understanding of good family support and the family's ability to provide comprehensive support can be a valuable resource for dealing with stress and critical situations and the burden caused by them. Accordingly Brivio et al. [66] reported that patients receiving higher levels of support from the family system and positive interaction with it, manage the crisis more efficiently and, therefore, experience more positive affect and less negative affect.

### Limitations

Because of the limitations of the study, the results should be interpreted with caution. These limitations include the relatively small size of the sample, using convenience sampling, selection of subjects from one hospital only, and specific cultural characteristics of the region, which may restrict the external validity of the study and the transferability of the results. Another limitation of the study is the fact that four questionnaires had to be completed simultaneously online on a self-report basis, which was time-consuming and tiring and might have affected the respondents’ answers. In addition, the collected data came from self-report questionnaires. It is suggested that future studies use other methods of data collection, including interviews. Another limitation of the study is its cross-sectional nature, which precludes definite conclusions and may subject the analysis of the results to retro-causality; thus, future studies may use longitudinal or interventional designs.

### Strengths

Despite the above-mentioned limitations, the findings of the present study are potentially important for the following reasons. First, only a few studies have explored the role of spiritual well-being and resilience and their correlation with COVID-19 anxiety in patients with cancer, who are especially vulnerable during the pandemic. Second, the findings of the present study can serve as a springboard for further evaluation of patients’ spiritual and religious needs and identification of other effective coping strategies that facilitate patients’ progress toward positive adaptation to their illness and, by extension, better mental health in the COVID-19 pandemic. Moreover, these findings can help healthcare policy makers develop and implement plans to improve cancer patients’ spiritual well-being and resilience as effective coping mechanisms in the face of cancer, especially during the COVID-19 pandemic. These findings also stress the need for training the healthcare personnel in providing patient-centered spiritual care on inter-disciplinary medical teams and promoting resilience in patients with cancer.

## Conclusion

In the present study, the COVID-19 anxiety, spiritual well-being, and resilience levels in the patients undergoing chemotherapy for cancer were average. In addition, the results showed a relatively significant direct correlation between the patients’ spiritual well-being and resilience and a significant inverse correlation between their spiritual well-being and COVID-19-related anxiety. In addition, there was a significant inverse correlation between the variables of resilience and COVID-19-related anxiety. Overall, the findings of the study confirm the major role of spiritual well-being in enhancing cancer patients’ resilience in battling their illness during the COVID-19 pandemic and, by extension, reducing their COVID-19-related anxiety. Accordingly, elevating cancer patients’ spiritual well-being and resilience must be seen as an integral part of their care and a valuable resource for fighting cancer and decreasing patients’ anxiety, especially during the pandemic caused by the coronavirus.

## Data Availability

The data that support the findings of this study are available from the corresponding author upon reasonable request.
